# How does the CNS control arm reaching movements? Introducing a hierarchical nonlinear predictive control organization based on the idea of muscle synergies

**DOI:** 10.1371/journal.pone.0228726

**Published:** 2020-02-05

**Authors:** Sedigheh Dehghani, Fariba Bahrami

**Affiliations:** CIPCE, Human Motor Control and Computational Neuroscience Laboratory, School of ECE, College of Engineering, University of Tehran, Tehran, Iran; Toronto Rehabilitation Institute - UHN, CANADA

## Abstract

In this study, we introduce a hierarchical and modular computational model to explain how the CNS (Central Nervous System) controls arm reaching movement (ARM) in the frontal plane and under different conditions. The proposed hierarchical organization was established at three levels: 1) motor planning, 2) command production, and 3) motor execution. Since in this work we are not discussing motion learning, no learning procedure was considered in the model. Previous models mainly assume that the motor planning level produces the desired trajectories of the joints and feeds it to the next level to be tracked. In the proposed model, the motion control is described based on a regulatory control policy, that is, the output of the motor planning level is a step function defining the initial and final desired position of the hand. For the command production level, a nonlinear predictive model was developed to explain how the time-invariant muscle synergies (MSs) are recruited. We used the same computational model to explain the arm reaching motion for a combined ARM task. The combined ARM is defined as two successive ARM such that it starts from point A and reaches to point C via point B. To develop the model, kinematic and kinetic data from six subjects were recorded and analyzed during ARM task performance. The subjects used a robotic manipulator while moving their hand in the frontal plane. The EMG data of 15 muscles were also recorded. The MSs used in the model were extracted from the recorded EMG data. The proposed model explains two aspects of the motor control system by a novel computational approach: 1) the CNS reduces the dimension of the control space using the notion of MSs and thereby, avoids immense computational loads; 2) at the level of motor planning, the CNS generates the desired position of the hand at the starting, via and the final points, and this amounts to a regulatory and non-tracking structure.

## Introduction

It is well-known that every joint is controlled by several muscles, each one having a specified role in motion generation at that joint. Therefore, the degrees of freedom (DoF) in the internal or muscular space of the human body is far more than the mechanical DoF that is used in the external or joint space; the latter is limited by the mechanical and anatomical constraints of the limb. Thus, on the one hand, the CNS benefits from an abundancy of possibilities in the internal space, which has to be structured and controlled. On the other hand, performance of any given movement in the external space is accomplished under several mechanical and anatomical constraints [1]. Therefore, Bernstein suggested that a hierarchical and modular organization does exist for realizing any movement properly [2]. Based on this picture, some researchers suggested that at the highest level of this hierarchical structure motor planning is performed, and then, at a lower level, the corresponding motor command is produced, and finally, at the lowest level, according to the musculoskeletal system of the limbs, the movement is executed [3,4]. Here, the key question is whether a computational model for controlling Arm Reaching Movements (ARMs) in the vertical (frontal) plane with the above hierarchical organization can be developed that employs modular structure based on recruitment of Muscle Synergies (MSs).

Many previous studies attempted to represent these three different levels in the computational models of motion control [5–13]. The first point we would like to take into consideration in these works is that all of these models are based on a tracking approach, that is at the level of command generation the desired motion trajectories, planned by the motor planning level, are tracked continuously. In this type of modeling approach, at the motion planning level, the desired trajectories that define the motion, *e*.*g*., trajectories of the joint angles, are determined through optimization procedures [9–15]. However, there are observations indicating that the control of ARMs has no tracking nature [16,17]. At least, based on these observations, we believe the CNS does not apply a continuous tracking control to execute ARMs.

Since the musculoskeletal system of ARMs has a complex and nonlinear characteristics but its performance is robust, it is believed that the CNS uses control methods at the command production level that are more matching to the above features. Different approaches were used to model the behavior of the CNS at this level: [18,19] used the impedance control, while [20,21] suggested an adaptive control strategy, [22,23] applied predictive control method, and [24,25] used optimal control methods in their computational models.

Another important question in this field is the question of control variables or motor commands: what is the variable used by the command generation level and send to the execution level. In previous studies, motor commands of computational models were produced at different levels, they could be joint torques [12,19], muscle forces [26,27], muscle activities [28,29] or even recruitment coefficients (RCs) of the motor primitives [30,31]. Since changes in the joint torques stem actually from changes in muscle forces, so we believe that computational models in which the motor command is produced at the muscle space can represent performance of the CNS more clearly. The concept of MS solves the problem of large amounts of computations, which is resulted from abundancy of the control variables, technically by reducing the dimension of control space through a modular organization [32–35]. Physiological evidences also confirmed the existence of MSs and suggested that at the motor execution level the effort to recruit MSs is minimized rather than the effort for controlling the individual muscles [36]. In this relation, different researches have suggested that it is possible to reconstruct patterns of muscles activities in ARM using the concept of MSs; for example following reconstruction approaches have been proposed and investigated: a combination of time-varying MSs [37,38], time-invariant MSs [34,39], or extraction of MSs based on co-contraction and reciprocal activities of the muscles involved in the task [40]. The important thing to note is that in all of these cases, although the dimension of control space has been reduced due to the use of MSs, the motor execution of ARM has been carried out as a tracking problem in the joint space.

**Fig 1:**
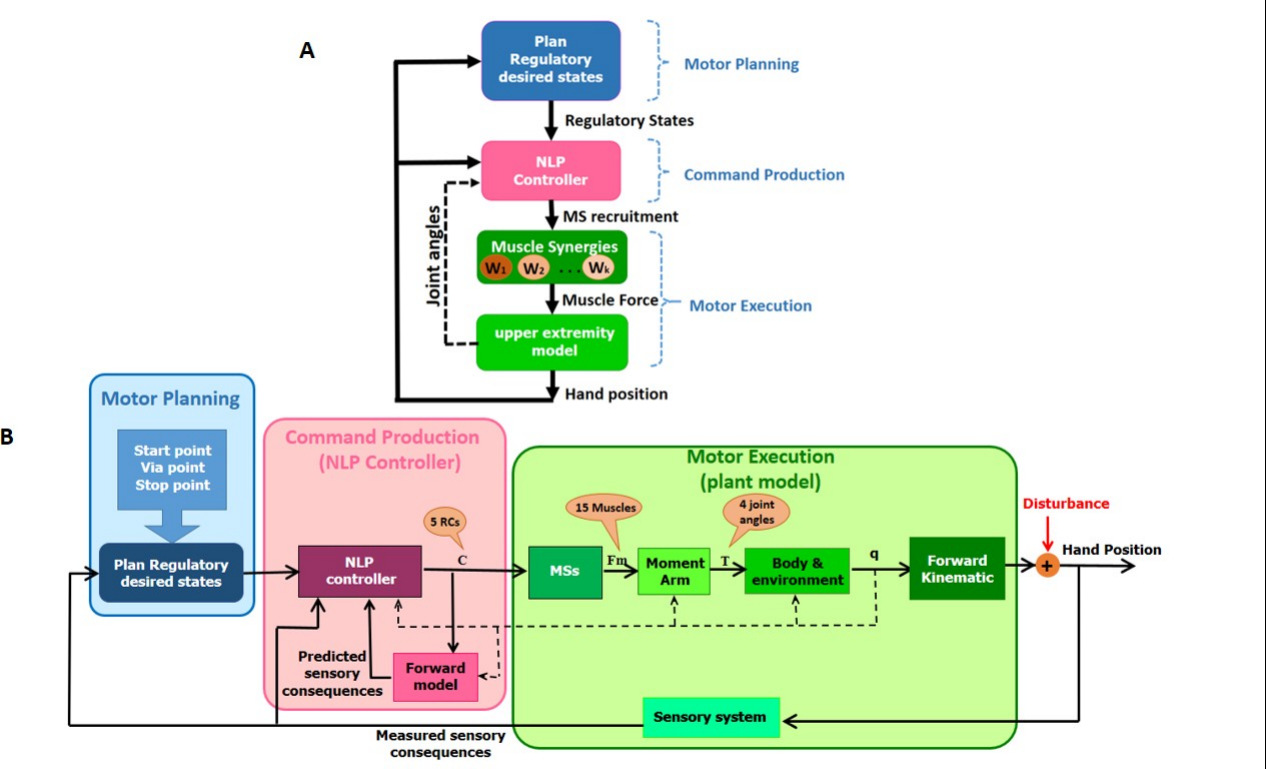
The proposed hierarchical and modular organization based on MS recruitment in ARM. A) hierarchical organization of three levels. B) the block diagram of the proposed computational model based on nonlinear predictive recruitment of MSs.

In this study, a new computational model is introduced to explain how the CNS controls ARMs using the idea of MSs. The proposed model introduces a hierarchical nonlinear predictive control organization for controlling ARMs under various constraints and in the frontal plane with the following features. First, it is developed based on a hierarchical and modular organization with three levels of motor planning, command production, and motor execution. Second, at the level of motor planning, the arm reaching movement is defined as a step function describing transition from the initial position to the final desired position. This corresponds to an input for a regulatory controller. This approach is generalized and used in the implementation and control of combined ARM. Third, at the motor command level, a nonlinear predictive controller (NPC) is used to recruit MS modules in a modular organization. As we assume a regulatory nature for the command production level, the control variables of the model are the RCs of the time-invariant MSs, which are determined by the nonlinear predictive controller. Finally, at the level of motor execution, the upper limb with three DoF in the shoulder joint and one DoF in the elbow joint (four DoF in the joint space) and 15 muscles (15 DoF in the control variable space) involved in the vertical ARM (corresponding to the hand movement on the frontal plane) are modeled in the musculoskeletal system. Fig 1A shows the general structure of the proposed hierarchical and modular model. The block diagram of the nonlinear predictive controller which is used to describe the recruitment of MSs is shown in Fig 1B. Three levels of motor planning, command production, and motor execution are well represented in this figure. The model is used to describe motor control of simple and combined ARMs in the presence and absence of external disturbances.

We also conducted a set of experiments to determine the kinematic and kinetic characteristics of simple and combined ARMs in the frontal plane. The participants interacted with a robotic manipulator designed in the Human Motor Control and Computational Neuroscience laboratory at the University of Tehran [41]. Then, MS modules were extracted from recorded EMG data. These synergies were used in the model.

Therefore, the structure of the paper is as follows. In the materials and method section, first we explain briefly the ARM experiments we performed, and then, the procedure to identify the corresponding MSs. Then the model of the motor execution level is introduced. It is consisted of the augmented musculoskeletal model together with the MS block. Next, the command production level is described. In this section, we explain how an NPC generates RCs of the MSs. Then, using the results of the experiments we show how the motor planning level generates the inputs of a regulatory control system for combined and uncombined ARMs. Finally, the performance of the proposed model is statistically analyzed and compared with the results of the experiments in the result section. We use the information of the hand position and the RCs of the MSs to do the comparison. In the last section we will discuss our results and validate the model.

## Materials and methods

### Experiments

**Fig 2:**
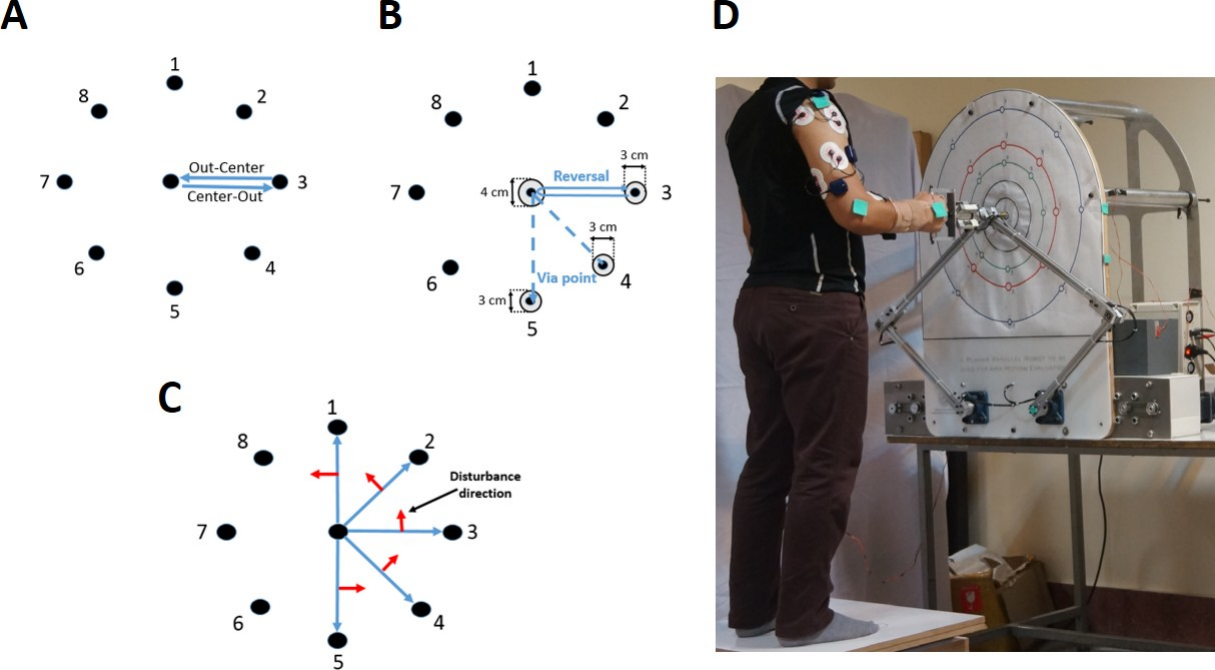
The reaching points on the frontal plane. A) simple motions, B) combined motions (dashed line: via-point motion, solid line: reversal motion), C) red arrow show direction of the applied disturbance force in the disturbed motions, D) A participant is standing in front of the robot and the EMG electrodes are attached on the 15 relevant muscles.

Experiments have been conducted to investigate MSs in simple and combined ARMs in the presence and absence of external disturbances. Eight points on a circle with a radius of 20 cm in the frontal plane were considered, where movements from these points to the center or vice versa were defined as “simple: sim” motions (Fig 2A). If the movement started from a point on the circle, then went to another point on the circle while passing through the 2 cm neighborhood of the center point, this combined movement was defined as “via-point: via” motion (Fig 2B). Also, if the points at the start and end of via-point motion were the same, this combined movement was defined as “reversal: rev” motion (Fig 2B). If in the middle of simple ARM, the hand of a person is moved unexpectedly out of his way in a perpendicular direction with an external unpredictable disturbance applied by the robot, this motion is defined as “disturbed: dis” motion (Fig 2C). Based on the protocol of the experiments with disturbances, during the implementation of a simple ARM, as soon as the hand position reaches a distance of 2 cm from the midpoint, the robot deviates the subject’s hand perpendicular to the direction of motion to a distance of 6 centimeters from the midpoint (as shown in Fig 2C). The midpoint is defined as the middle of the line connecting the start and stop points.

Each movement is evaluated by the robot according to the items in the ‎Table 1. If these are not approved, the movement will be repeated again. Movement duration definition is described in the ‎S1 File.

**Table 1:** Items evaluated by the robot to consider a trial as acceptable. When the constraints were not met, the trial was rejected and the subject had to repeat the trial.

Motion type	Movement duration	deployment at End point	Accepted neighborhood for			
			Strat point	Via point	End point	Mid-point
**via**	< 800 ms	> 1s	< 1.5 cm	< 2 cm	< 1.5 cm	-
**sim**	< 400 ms	> 1s	< 1.5 cm	-	< 1.5 cm	-
**dis**	< 400 ms	> 1s	< 1.5 cm	-	< 1.5 cm	< 2 cm

In all tests, the wrist joint is fixed with a splint, and the person can only use three DoF in the shoulder joint and one DoF in his elbow by keeping the handle of the robot (Fig 2D). The start, via and target points are announced through the audible message from the examiner to the subject. Six healthy male volunteer subjects in the age range of 25.7 ± 1.3 years, with no history of musculoskeletal and neuromuscular diseases participated in these experiments. The height of the participants was 175.8 ± 3.1 cm and their weight was 76.17 ± 8.37 kg. All subjects were right-handed, and they were asked to perform ARMs with their right hand. Subjects randomly repeat each possible type of ARMs (16 sim, 16 dis, 56 via and 8 rev) for five times. The subject was instructed to rest for one minute after each single trial (movement) to recover from tentative muscle fatigue during motion execution. The experimental protocol was approved by the Human Research Ethics Review Committee from Iran University of Medical Science. Also, a verbal type of informed consent was obtained from the subjects.

### Identification of the MSs

As the proposed computational model utilizes a modular organization based on MSs, it is required to identify MSs. For this purpose, kinematic and muscular activity data of combined and uncombined ARMs has been measured in the presence and absence of external disturbances during experiments. In this study we used time-invariant MSs. These time-invariant modules (which are also named spatial synergies) are muscles activated in synchrony with fixed relative gains as reported in [42,43]. Thus by recruitment of a few spatial MSs, the coordinated spatiotemporal muscle activation patterns observed during ARM could be reproduced. Here, the nonnegative matrix factorization (NMF) algorithm was used to extract MSs as time-invariant modules [44,45] for each subject. The experiment protocol is described in S1 File and showed in S1 Fig. The Variance Accounted For (VAF) criterion with a threshold level of 90% has been used to determine the number of MSs [37,45–52]. Acording to the results of our experiments, the number of MS vectors to reconstruct the muscle activities with a VAF value of 90% for each subject is represented in ‎Table 2.

**Table 2:** The number of MSs’ vectors to reconstruct muscle activity with a VAF value of 90% for each subject.

	Subject 1	Subject 2	Subject 3	Subject 4	Subject 5	Subject 6
Number of MSs	5	7	3	7	5	9

The similarity index of Correlation Degree Measure (CDM) is used to compare MSs recruited in different types of ARMs (Eq 1). Thus, if the set W_1_ consists of n_1_ MSs, and the set W_2_ has n_2_ MSs, then, the CDM similarity index between these two sets of MSs is given as follows:

$$CDM:S(W_{1},W_{2})=\frac{1}{n_{1}}\sum_{i=1}^{n_{1}}max[\rho (W_{1i},W_{2j}){|}_{j=1}^{n_{2}}]$$ (1)

Where ρ is the Pearson correlation coefficient. We extracted the MSs (VAF>90%) of each type of movement (simple, via, disturbed trials) for each subject separately. Then, for each subject the similarity index (CDM) among the tasks, under different conditions, were evaluated. The average value of CDM among the tasks for each subject is presented in ‎Table 3. In this case, the CDM of the extracted MSs over the six subjects (average of the data in ‎Table 3) is 0.92 ± 0.02. Our experimental results show that similar MSs are employed in radial movements under various constraints, that is, the MSs extracted in simple motions are similar to those in disturbance and via-point motions.

**Table 3:** Evaluation of the similarity index (CDM) in extracted MSs of different conditions for each subject.

	Subject 1	Subject 2	Subject 3	Subject 4	Subject 5	Subject 6
**CDM**	0.94±0.02	0.93±0.01	0.91±0.04	0.94±0.03	0.93±0.05	0.91±0.02

The experimental results showed that the extracted time-invariant MSs in performing various simple movements (W_s_) were similar to those MSs extracted in combined movements (W_c_) and in the presence of external perturbation (W_d_) for all subjects (‎Table 4).

**Table 4:** CDM of MSs for different types of movements for all subjects.

	S_Ws,Wc_	S_Ws,Wd_
**Mean**	0.9330	0.9458
**STD**	0.0063	0.0335

Thus, with respect to ‎Table 3 and ‎Table 4 in the proposed model, the MSs in the implementation of combined and disturbed movements is considered as the MSs extracted from simple ARMs.

### Motor execution model based on MSs

**Fig 3:**
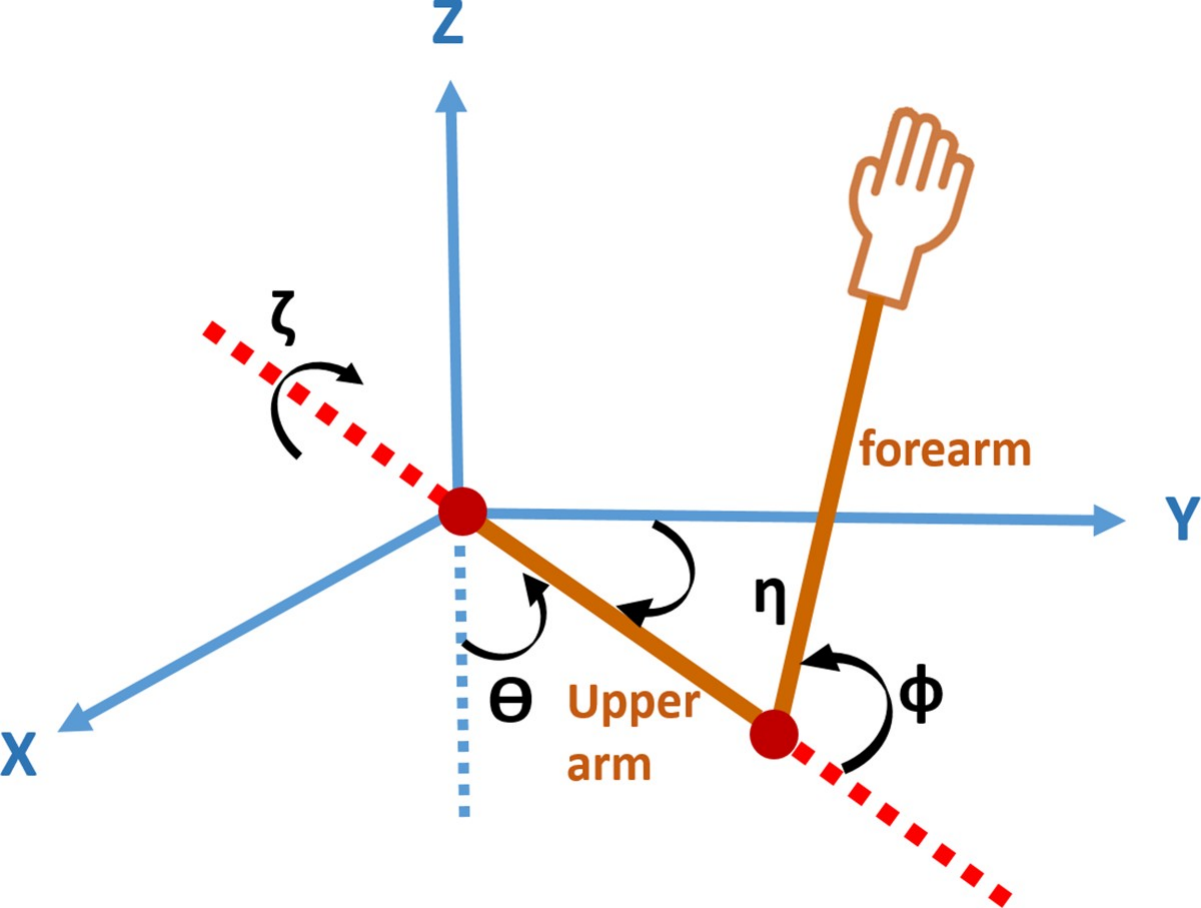
Joint angles of human arm with three joint angles in shoulder and one joint angle in elbow.

Based on conducted experiments, and at the motor execution level of the hierarchical organization of the proposed model, it is necessary to consider a musculoskeletal structure for the upper extremity with four joint angles. In the proposed model, two DoF in the wrist and supination/pronation motions of the elbow joint were assumed fixed. Thus, the structure of the arm can be expressed using four joint angles (‎Fig 3) via the following equation:

$$q≔{\left(\theta ,\eta ,\zeta ,\phi \right)}^{T}\in Q$$ (2)

In this equation ***q*** indicates four DoF in the upper extremity. Where *θ* is the elevation angle, *η* is the azimuthal angle and *ζ* represents the humeral angle of the shoulder joint, and finally, *ϕ* represents the flexion of the elbow joint. In general, the dynamic equations of the upper extremity can be written with these four joint angles as follows:

$$I(q)\ddot{q}+V(q.\dot{q})+J^{T}F_{ext}=\tau \to \ddot{q}={I(q)}^{-1}\{\tau -V(q.\dot{q})-J^{T}F_{ext}\}$$ (3)

In the above equation, *I* is the inertial matrix, $V(q.\dot{q})$ is the summation matrix of two Coriolis and gravitational torques, *J* is the jacobian of the arm, *F*_*ext*_ is the external force of the environment, and ***τ*** is the torque produced in the joints.

The robot used in our experiments is a 5 link parallel robot designed and evaluated in the human motion control lab of the school of the electrical and computer engineering at the University of Tehran [41] to assess the movements of the upper limb. A control system is used to compensate its load (or more precisely, its dynamics) when interacting with the subjects. For this reason, we assumed that the external force of the robot on the subject’s hand in simple and combined ARMs is zero.

According to [53], the elements of the above matrices are calculated based on the anthropomorphic characteristics of each individual. To simulate the proposed model, we used the MSs extracted for each subject individually; in other words, we ran the simulation for each subject separately. However, for the sake of briefness, only the results of one typical subject are reported, although similar results have been obtained for other subjects and for the simulation of other motion directions. The model proposed in this research has been computed for subject 5 with the following anthropomorphic characteristics (‎Table 5).

**Table 5:** Anthropomorphic characteristics of subject 5 based on [54].

	m_i_ (Kg)	I_i_,_x_ ($Kg\frac{m^{2}}{s^{2}}$)	I_i_,_y_ ($Kg\frac{m^{2}}{s^{2}}$)	I_i_,_z_ ($Kg\frac{m^{2}}{s^{2}}$)	L_i_ (m)	r_i_ (m)
**i = u: upper arm**	1.56	0.0079	0.0079	0.0029	0.3013	0.1216
**i = f: forearm**	1.52	0.0299	0.0299	0.0013	0.4082	0.4082

The parameters m_i_, I_i_,_x_, I_i_,_y_, I_i_,_z_, L_i_, r_i_, (i = u, f) denote mass, principal moments of inertia around the x-, y-, and z-axes of the body-fixed coordinate systems, length, and distance to the center of mass of the upper and forearm, respectively [54]. It was assumed that the (transversal) x and y components of the moment of inertia for the upper arm and forearm, respectively, are the same; i.e., I_u_,_x_ = I_u_,_y_ and I_f_,_x_ = I_f_,_y_.

According to the conducted experiments, 15 upper extremity muscles are considered in the introduced model. To generate the desired torque to change the three joint angles in the shoulder and one joint angle in the elbow, it is necessary to determine the moment arm of each muscle during movement. The moment arm value for each of these 15 muscles, has been defined as a polynomial function of appropriate joint angles leading to its movement (details have been described in S2 File). It is worth noting that providing the moment arms of these 15 muscles in an integrated way is one of the innovations of this research. Using the *M*_*arm*_ matrix and having the force of each muscle (***F***_***m***_), the torque generated at each joint can be calculated as follows.

$$\tau =M_{arm}\times F_{m}\to \ddot{q}={I(q)}^{-1}\{M_{arm}\times F_{m}-V(q.\dot{q})\}$$ (4)

The muscle forces are considered as a result of multiplication of MSs in the matrix of RC. Matrix decomposition based on the NMF method is used to extract MSs of muscle force data (as described in the S1 File). Thus, the force of muscles involved in the motion can be shown by employing weighted recruitment of ***k*** MS vectors. In this case, the MS vectors are placed in the ***W*** matrix (of ***ch*** = **15** channels) and the time-variant matrix ***C***(***t***) represents the RC of these MS vectors.

$$F_{m}=W_{\left(ch\times k\right)}\times {C(t)}_{\left(k\times samples\right)}\to \ddot{q}={I(q)}^{-1}\{M_{arm}\times (W\times C)-V(q.\dot{q})\}$$ (5)

### Applied perturbation

Based on the protocol of the experiments with disturbance, during the implementation of a simple ARM, as soon as the hand position reaches a distance of 2 cm from the midpoint, the robot deviates subject’s hand perpendicular to the direction of motion to a distance of 6 centimeters from the midpoint (as shown in Fig 2C). We implemented exactly the same procedure in our model and after introducing the disturbance, we deviated hand position 6 cm in a direction perpendicular to its movement direction. We defined this 6 cm deviation, as the disturbance intervened by the robot.

### Nonlinear predictive approach in recruitment of the MSs

It is suggested that in performing fast and skillful movements, a proper motor command is produced according to the internal model prediction in the cerebellum [55,56]. The musculoskeletal system, in general, has complex nonlinear dynamics. Therefore, we think that a nonlinear predictive method (similar to what is proposed in [57]) can describe more appropriately the behavior of the CNS when producing motor commands. In order to implement this method computationally, it is necessary to determine the state variables of the musculoskeletal system.

$$X_{1}=q=\left[\begin{array}{c} \theta \\ \eta \\ \zeta \\ \phi \end{array}\right],X_{2}=\dot{X_{1}}=\left[\begin{array}{c} \dot{\theta }\\ \dot{\eta }\\ \dot{\zeta }\\ \dot{\phi } \end{array}\right]\to X=\left[\begin{array}{c} X_{1}\\ X_{2} \end{array}\right]=\left[\begin{array}{c} \theta \\ \eta \\ \zeta \\ \phi \\ \dot{\theta }\\ \dot{\eta }\\ \dot{\zeta }\\ \dot{\phi } \end{array}\right]$$ (6)

The state space equations of this nonlinear musculoskeletal system is as follows.

$$\dot{X_{1}}=X_{2}=f_{1}(X)=q$$ (7)

$$\dot{X_{2}}=\ddot{X_{1}}=f_{2}(X)+B_{2}(X)u={-I(q)}^{-1}\{V(q.\dot{q})\}+{I(q)}^{-1}\{M_{arm}\times W\}u$$ (8)

y=H(X) (9)

Where y, the hand position, is calculated using H as a function of joint angles according to [48]. Subsequently, the NPC attempts to minimize the amount of effort and the position error for the nonlinear musculoskeletal system along the prediction horizon of *h*, by using Lie derivatives. The NPC cost function is as follows.

$$J[u(t)]=\frac{1}{2}{\left[y(t+h)-y_{d}(t+h)\right]}^{T}Q[y(t+h)-y_{d}(t+h)]+\frac{1}{2}{u(t)}^{T}Ru(t)$$ (10)

Where *u*(*t*) is the NPC’s output that determines the RCs of MSs. Also, *y*_*d*_(*t*+*h*) represents the desired hand position for *h* moments later. *Q* and *R* are the diagonal matrices as two design parameters for weighting the position error and the amount of input effort, respectively. Since in the NMF method the decomposed MSs and their coefficients were assumed to be non-negative, here the command generated by the NPC is constrained to be non-negative, too. The minimization of the constrained cost function leads to the production of a motor command of the RCs of MSs.

### NPC parameter setting

According to references [58,59], if a large value is chosen for the lower limit of the prediction horizon (h), it means that the error is not important in the early times and this will slow down the transient response. On the other hand, this limit has no effect on the optimization procedure if it is less than the system delay. In stable systems, the open loop system settling time is considered to be the upper limit of h. The reference [60] states that the value of h must be chosen to be large enough such that its magnification does not affect the control signal. Based on these facts, the prediction horizon for the proposed model is chosen to be 10 time samples (10 mili seconds). The time interval of the simulation was 400 mili seconds, too.

Weighting matrices (Q & R) are the most influential parameters in the criterion of stability, performance, and robustness of the closed loop system [61]. Weighting matrices play role in scaling prediction errors and control signal variations, preventing ill-conditions in calculating control signal integrals, and limiting control signals. In the proposed model, the values for the weighting matrices in the cost function are determined based on the analysis of variance of the measured values of the hand position and the amount of muscle activity in the experiments coefficient [62]. Thus, the non-zero elements of *Q* and *R* matrices were 10^8^ and 1, respectively.

In the proposed model we used the concept of time-invariant MSs’ recruitment in a hierarchical and modular structure while applying a nonlinear predictive procedure, hence, we assumed that there is no physiological sensory feedback delay. Therefore, one of the questions that will be the subject of our future research is the effect of sensory feedback delay on the performance of the model.

### ARM regulatory motor planning

**Fig 4:**
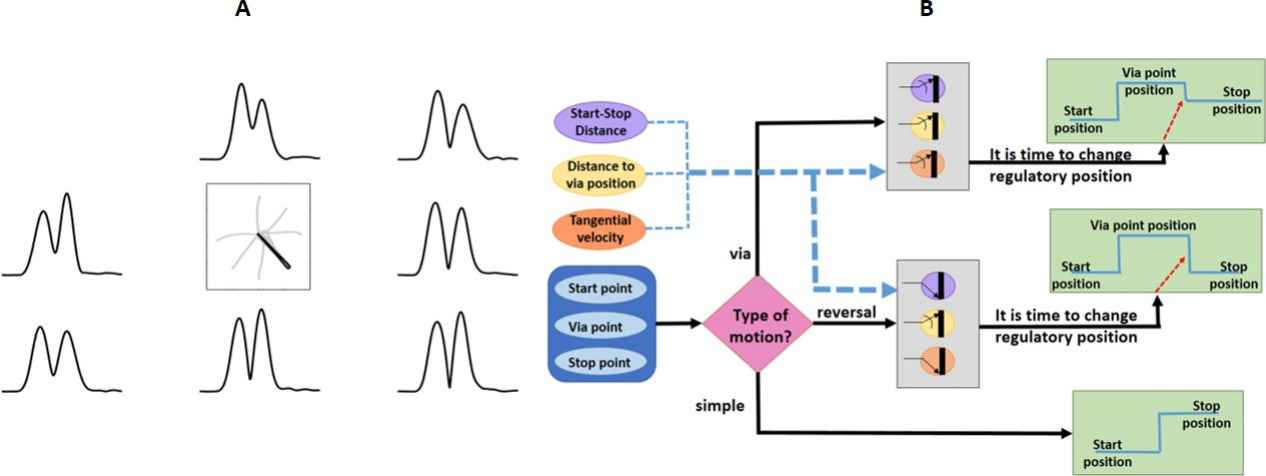
The effect of kinematic information on the motor planning level. A) The tangential velocity of the hand while approaching the center is closer to zero when moving from point 4 to points 3 and 5, and the tangential velocity is not zero in the movements with the final points 1, 2, 6 and 7. In addition, the tangential velocity at the center in reversal movement from point 4 to point 4 is exactly zero. B) the block diagram of decision for states of the desired input for ARMs in all directions.

In the production of motor command by NPC, it is necessary to minimize the error of the hand position at each moment from the final desired position. What is considered in the simple ARM implementation (‎Fig 2A), is moving from one starting position to the final position. In the model presented in this study and with respect to the starting and final points of the movement, the regulatory nature of the motor planning is described by applying a step input which causes movement from the initial position to the desired position. We used the result of our data analysis to develop the input (generated by the motor planning level) to the command production level for combined ARMs. According to our observations, to perform via-point or the combined movements, the hand changed its direction toward the second point (the final goal) when it laid in a neighborhood of the via-point with radius of 2 cm. On the other hand, according to the results of the experiments, whenever the start and stop points of the combined movements were closer to each other than to the via-point, the tangential velocity at the via-point was closer to zero when passing through the 2 cm neighborhood of the via-point (‎Fig 4A). This shows that in the implementation of the combined ARM, the tangential velocity of the subject's hand when passing through the via-point depends on how the start and stop points are positioned relative to each other. Therefore, at the level of motor planning of the proposed model, the time for changing the desired regulatory input is determined according to three parameters: 1) the hand position, 2) the amount of proximity to the via-point, and 3) the tangential velocity of the individual's hands (Fig 4B).

### Statistical analysis to validate the model

Here, the regression analysis has been used as a powerful statistical method to examine the relationship between the simulated hand trajectories and the distribution of the experimental trajectories. The regression equation tries to find the best fit line for the simulated hand trajectory with the help of the experimental one as follows [63].

$$P_{exp}=P_{sim}+\varepsilon$$ (11)

Where, *P*_*exp*_ and *P*_*sim*_ represent the experimental and simulated hand trajectories, respectively. Also, *ε* represents the regression residue. The mean squared residue is identified as the statistical p-values of regression analysis to determine whether the relationships that we observe in the experiments also do expressed by the simulated model. Therefore, if the p-value is less than the significance level (which is chosen 0.05), then we conclude that the behavior of the proposed model fits the experimental data properly.

In order to compare the RCs of MSs, their time- and frequency-domain features have been used. Therefore, Mean Absolute Value (MAV), Modified Mean Absolute Value 1 (MMAV1), Modified Mean Absolute Value 2 (MMAV2), Mean Absolute Value Slope (MAVS), Root Mean Square (RMS), Variance of RCs (VAR), Waveform Length (WL), Zero Crossing (ZC), Slope Sign Change (SSC), Willison Amplitude (WAMP), and Simple Square Integral (SSI) have been used as 11 time-domain features. Also, Median Frequency (FMD), Mean Frequency (FMN), Modified Median Frequency (MFMD), Modified Mean Frequency (MFMN), Frequency Ratio (FR) were used as five frequency-domain features. All of the above features had been defined in reference [64]. Then, each RC was divided into 10 windows, and the values of 16 features were calculated at each interval. Finally, the feature matrix for each of RCs is a matrix with 16 rows and 10 columns. Those columns represent the feature values for ten intervals. It is worth mentioning that all features were normalized before checking similarities. The two-dimensional correlation criterion (corr2) has been used to compare these feature matrices.

## Results

**Fig 5:**
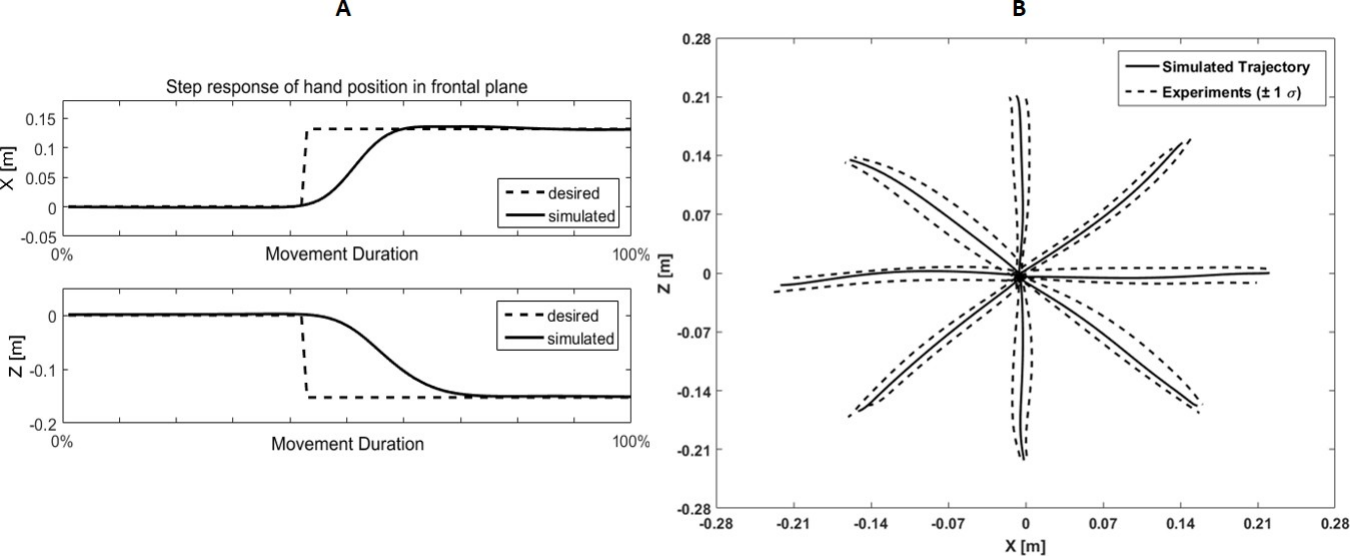
Simulated hand position in simple movement. A) X and Z coordinates in simple movement from the center to point 4 in the frontal plane. B) Hand trajectory in the simulated model with NPC (solid lines), the range of changes in hand trajectories in experimens (dashed line).

**Fig 6:**
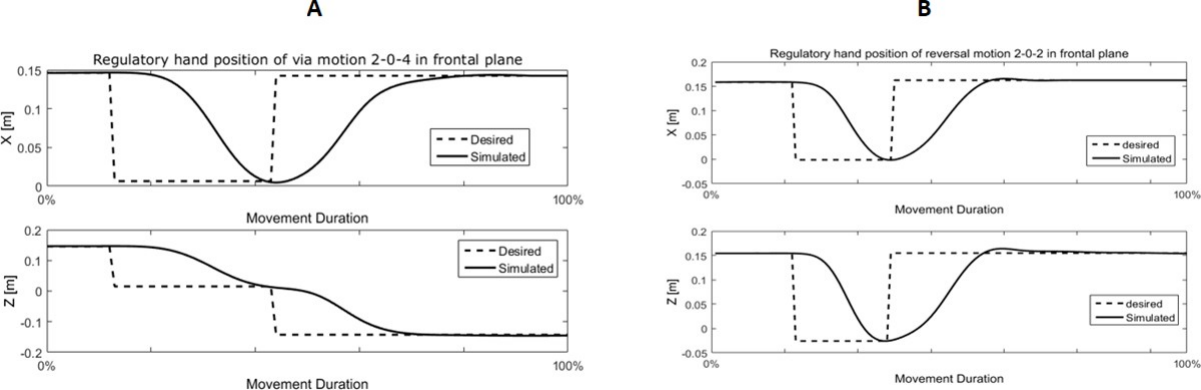
Simulated hand trajectory in combined motions. A) via-point motion from point 2 to point 4 in frontal plane, B) reversal motion from point 2 to point 2 in frontal plane.

Since the implementation of different types of ARM is considered in this research, it is necessary to evaluate the performance of the proposed model in the execution of these movements. The simulation result of the proposed model in the implementation of a simple ARM from the center to point 4 has been presented in ‎Fig 5A. Simulated hand trajectory from the center to all eight points with respect to the range of variations of hand trajectory in repeated trials of experiments has been shown in ‎Fig 5B. Also, simulated combined ARM from point 2 to point 4 by passing the center has been illustrated in ‎Fig 6A. Simulated reversal motion from point 2 to the center and returning back to point 2 has been shown in Fig 6B.

**Fig 7:**
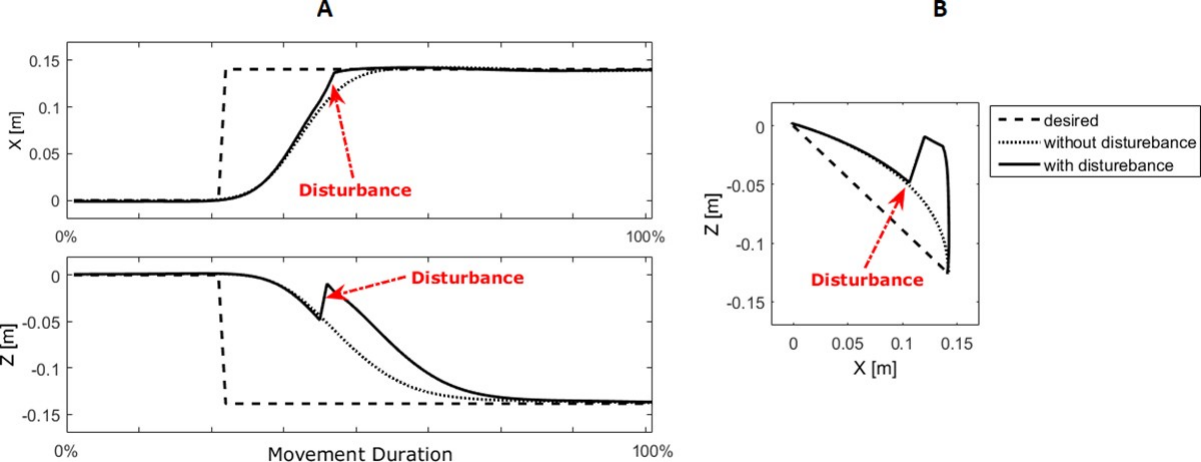
Simulated hand trajectory in simple disturbed motion from the center to point 4 in frontal plane. Dashed line: desired regulatory state, dot line: without disturbance, solid line: disturbed simulated motion. A) X and Z coordinates. B) hand trajectory.

According to the experimental results, MSs in the presence of disturbances were the same as MSs used in the implementation of non-disturbed movements. Unpredictable disturbance in the model is applied according to the protocol of the experiments. Thus, to simulate any ARM with disturbance, during the implementation of a simple ARM when the hand is in a distance of 2 cm from the midpoint, a force perpendicular to the direction of motion of the subject’s hand is applied to deviate it to a distance of 6 centimeters from the midpoint. For implementing the disturbance in simulation, we changed the hand position to the perturbed position indicated in ‎ Fig 2C with the red arrow as soon as the hand position reaches a distance of 2 cm from the midpoint. The performance of the proposed model against unpredictable disturbances in the midpoint of simple ARM from the center to point 4 is presented in Fig 7.

In order to compare the output of the model with the experimental data, or in other words to validate the behavior of the model, the simulated hand trajectories are statistically compared with the distribution of the experimental trajectories using regression analysis of Eq 11. The results are represented in ‎Table 6.

**Table 6:** Regression analysis performed to compare the simulated hand trajectories and the experimental trajectories for each subject separately. Statistical significance is considered at the level of P-value<0.05.

	Subject 1	Subject 2	Subject 3	Subject 4	Subject 5	Subject 6
**Simple**	0.013	0.018	0.015	0.026	0.011	0.012
**Disturbed**	0.021	0.021	0.024	0.011	0.020	0.036
**Via-point**	0.015	0.016	0.026	0.026	0.018	0.016
**Reversal**	0.019	0.015	0.016	0.011	0.021	0.021

**Fig 8:**
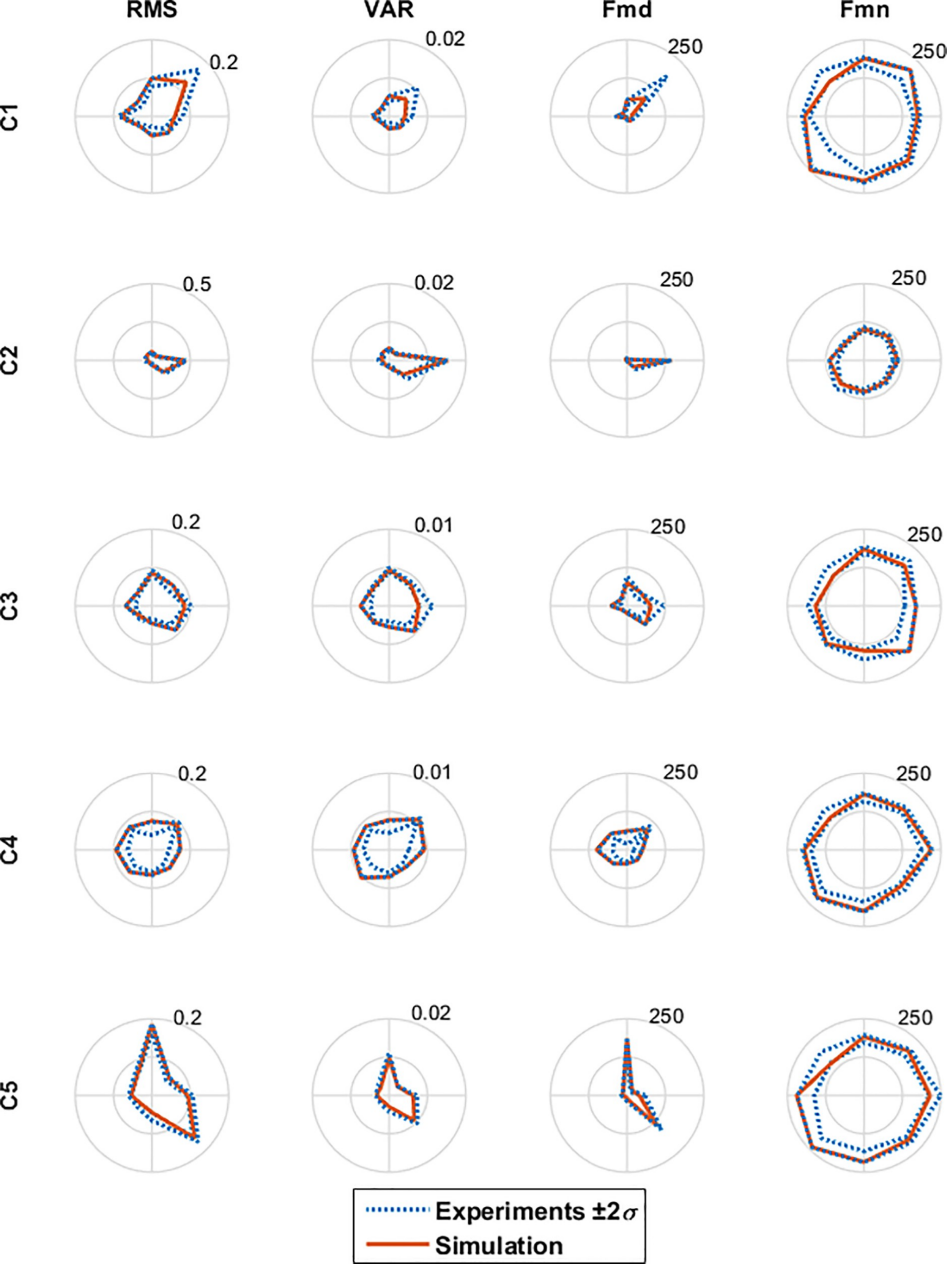
Four features of simulated RC of MSs in all eight center-out ARMs with respect to the RCs’ feature variations in experiments. As the subject #5 had five MSs, there exists five RCs (C1 to C5). The Features are Root Mean Square (RMS), Variance (VAR), Median Frequency (FMD), and Mean Frequency (FMN) of RCs.

**Fig 9:**
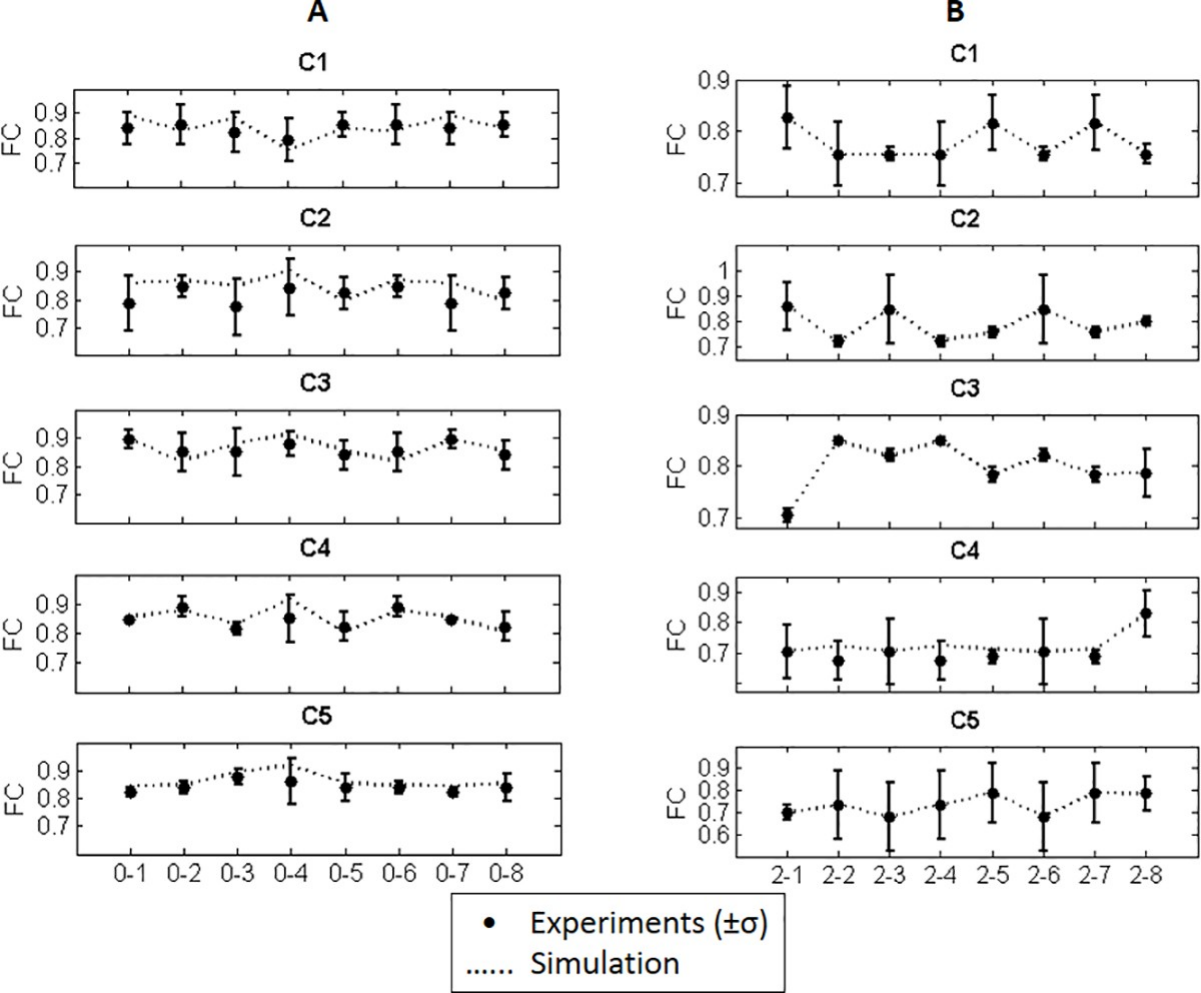
The variation of FC of RCs in the experiments (solid line) with respect to FC of simulated RC and average RC A) in all eight center-out simple ARMs, B) in all combined ARMs in frontal plane that start from point 2.

In addition to hand trajectories, it is necessary to compare the RCs of MSs with the values obtained from the experimental measurements, in order to evaluate the computational model’s performance. Given that in the experiments, every ARM is repeated five times randomly, the simulated RCs can be compared with RCs obtained from experiments. For example, four features (RMS, VAR, FMD and FMN) of the RCs of MSs in all eight center-out simple ARMs and the range of their variations in five repetitions of the same movements in the experiments is shown in Fig 8. The mean and standard deviation are made across the five different repetitions of the same task in the experiments. Given that every movement is repeated five times randomly, the mean value and standard deviation of feature matrices correlation in various repetitions for all eight center-out ARMs without disturbances are shown in Fig 9A as the solid line. Also, the Feature Correlation (FC) of RCs obtained from the simulation is indicated as the dashed line in Fig 9A.

The FC value of RCs in all combined motions that contains simple ARM from the center to point 2 as their first part of the motion with respect to their variations measured in the experiments is shown in ‎Fig 9B.

In order to compare the RCs derived from simulation of the model with the mean value of RCs of different iterations of the same motion in the experiment, the correlation of their feature matrices is calculated. The percentage of the similarity for the above comparison for all subjects is presented in ‎Table 7.

**Table 7:** The percentage of the mean similarity for the feature matrices of simulated RCs and the mean value of RCs of different iterations of the same motion in the experiments.

Motion	Subject 1	Subject 2	Subject 3	Subject 4	Subject 5	Subject 6	All subjects
**Simple**	87.29	89.94	89.68	89.51	90.3	84.94	88.61±1.91
**Disturbed**	85.73	85.47	80.31	85.66	79.64	83.54	83.39±2.53
**Via-point**	85.27	84.64	85.57	85.61	85.89	86.29	85.54±0.51
**Reversal**	87.1	84.64	85.57	89.61	85.89	83.05	85.97±2.04
**Mean**	86.34±0.86	86.17±2.20	85.28±3.32	87.59±1.96	85.43±3.79	84.45±1.26	
**P-value**	0.03	0.02	0.04	0.03	0.03	0.04	

As illustrated in ‎Table 4, the similarity index of feature matrices of RCs, for the simulation and experiments for all motions and all subjects, is 85.88±1.86% (P-value < 0.05).

## Discussion

The results of the simulations (‎Figs 5 and 6) show that the hierarchical and modular organization of the recruitment of MSs based on NPC can properly generate the hand position within the range of variations of the measured experiments. According to ‎Table 6, the p-value of the regression analysis of the simulated hand trajectory were lower than the acceptance threshold (p-value < 0.05). Therefore, it is reasonable to hypothesize that the simulated hand trajectories are statistically similar to the experimental trajectories of all subjects.

On the other hand, RCs of MSs derived from the simulation were in the range of changes obtained from the experiments (and 9). The statistical analysis reported in ‎Table 7, shows that the RCs resulted from the proposed model are similar to the RCs derived from the experimental data with a similarity value of 85.88±1.85%. These suggest the proper performance of the proposed computational model in the implementation of simple, combined, and reversal ARMs in the external (hand position) and internal (muscle force) spaces with respect to the experiments.

In the presence of disturbance, the CNS attempts to perform ARM in a way to correct hand trajectory so that it can reach the target point more accurately while minimizing the effort of MSs’ recruitment. Therefore, after applying the disturbance, the controller must correct the hand position so that it continues toward the target. As shown in Fig 7, by applying the disturbance, the hand trajectory has actually changed, and the controller corrected the position of the hand that went out of the normal trajectory by returning it to the nearest point of the movement trajectory. This is consistent with the results of the experiments carried out in this study (‎Table 6).

As our results indicate the behaviors of the proposed model in many different aspects are similar to the motor behaviors of our participants during ARM experiments. In this section, we also compare the behavior and structure of the proposed model (at three levels) with those of other models developed previously; and finally, we discus physiological evidence for the proposed model.

### Motor planning

Previous studies have suggested different cost functions in the optimization process when generation the desired motor behavior (input of the motor command production). Some of these cost functions to be mentioned are: maximization of task achievement [14], minimization of the jerk [9] or variance of the final position [10], minimization of torque changes [11] or commanded torque changes [15], and a combination of the above criteria [12,13,65,66]. Some scholars have discussed how to implement these cost functions in biological networks [67]. It is important to note that in these works, the control process (command production) is performed according to a tracking format in the joint space, and the form of movement is pre-planned according to the optimization criterion and the dynamic equations of the motor system. The only model we found that has considered the effect of sensory feedback in the presence of noise on the shape of the arm reaching velocity was presented by Todorov and Jordan [25]; otherwise, in other models developed based on optimization methods, the effect of the environment or the sensory feedback on the desired movements are not considered.

As mentioned before, there are observations indicating that arm reaching movement is controlled based on a regulatory strategy [16,17]. Therefore, we stayed at this finding and used a simple step function for simple ARM cases to describe the input of the second level or in other words to show the desire for changing the state or position of hand from its initial state to the final one.

Furthermore, in combined movements, the desired regulatory input is planned based on the position of the start, stop and via points, and hand tangential velocity. Also, this desired regulatory state is corrected according to the sensory information of the movement. For this purpose, it is necessary to allocate weights to each of the two decision criteria, which are the distance from the via-point and the tangential velocity of the hand at this point. Then, according to these weights, the motor planning level can make the right decision about the time when the regulatory input of the second level changes its value from the value indicating the via point to the value indicating the final state (‎Fig 4B).

### Motor command production

On the other hand, due to the abundance of DoF in the internal space at the motor command production level, that is the abundancy in the number of muscles compared to the DoF at the joints, the production of motor commands using individual muscles makes the control process complex and costly. Some researchers stated that the CNS would probably try to simplify the control process and computations by reducing the dimension of controlled variables using a modular organization [68–72]. In these frameworks, the introduced modules were composed of internal models in the mosaic structure [66], dynamic responses [73], internal models in each phase of the movement [7], eigen movements in the joint space [74], time-varying MSs [37,38], time-invariant MSs [34,39], or extraction of MSs based on co-contraction and reciprocal activities of the muscles involved in the task [40]. In all of the mentioned studies, although using modular organizations has reduced the complexity of movement control, it is still performed in the framework of tracking the desired joint trajectory.

Some researchers have implemented a recurrent neural model of the spatiotemporal MSs using the interaction of three core synergistic systems: a response generation system (R), a selector system (S) and a control system (C) to generate movement [75]. Although they introduced a hierarchical modular structure, they have used a simple 2- jointed arm model with 6 muscles. Also, they have modeled the spatiotemporal MSs with recurrent neural networks, and controlled human arm movement by activating three pairs of agonist/antagonist muscles.

Another group of researchers [76] used iterative linear quadratic regulator (iLQR) method to calculate optimal muscle activation required for a 2-link 6-muscle human arm moving in the horizontal plane. They used a variant of NMF developed by d’Avella et al. [77] to extract spatiotemporal synergies of the matrix of optimal activation level computed by the iLQR for a given task. Although their model was 10-dimentional (2 joint angle, 2 joint velocity, 6 activation), they extracted 6 MSs (equal to the number of involved muscles), and stated that optimal movements can be planned in a relatively low-dimensional space by time-shifting and linearly combining a small number of synergies.

In this work we used time-invariant MSs to control ARMs. Therefore, the MSs constitute the modular organization of the computational model. Here, the required motor command is produced by the NPC with respect to the present and future states of the system. These commands are the RCs of MSs. Since in this work we are not concerned with the learning procedure, therefore, the structure of MSs does not change during the movements. Five pre-determined MSs together with their RCs generate 15 muscular forces needed to perform the movement. This shows how using the modular structure developed based on the idea of MSs can reduce the DoF in the control space and simplifies the control process for the CNS. These results are in agreement with [33,70,72,78]. Furthermore, the hand position in the frontal plane will change due to the changes in the joint angles through applying the force of the muscles.

### Motor execution

In fact, there are researches about ARMs in the horizontal plane under the constraints that both the shoulder and elbow joints has only one DoF [26,79,80]. Therefore, 6 to 8 muscles are involved in these studies. However, in a reaching activity in which 3 DoF of the shoulder joint is use, at least 15 muscles are involved. In these studies, a support usually is used to compensate for the arm weight. Therefore, in such cases the complexities in arm motion control due to weight compensation and overcoming the torque caused by the gravity are not present. Whereas in the presented model, the arm moves in the three dimensional space while the hand is moving on the frontal plane (horizontal as well as vertical directions). Obviously, this type of movement is more in conformity with arm motions during daily activities. Also, due to the authors’ best of knowledge there has been no integrated model for calculating the moment arm of these 15 muscles. While in the presented model, the above mentioned features have been considered as the other innovations of this research.

### Physiological evidence of the proposed model

It was shown that CNS assigns different values to different options and for the stimuli it receives from the environment [81,82]. That is, the CNS executes motion with the highest accuracy and the lowest metabolic cost in terms of rewards and costs. Previous studies showed that in the execution of movements what is minimized by the CNS is not the amount of effort required by each muscle, but more exactly, it minimizes the amount of effort exerted by MSs during movement [36]. This fact is well seen in the cost function equation of the NPC (Eq 10) in the proposed model.

It is known that in ARM the subject tries to move his hand so that the distance to the target point is minimized. Since ARM in daily activities is carried out as a ballistic and skillful movement, the execution of these movements with tracking the desired trajectory leads to slow motions (due to the continuous use of feedback data). Inspired by the equilibrium point hypothesis, the time profile of set-points may be referred to as the equilibrium trajectory of the system [83]. The regulatory motor planning of the proposed model by specifying some states of the trajectory provides an elegant explanation of postural regulation, reported in [84]. Therefore, it is not required to track any information about the joint trajectory fed into the controller as the desired input, and also the complex trajectory planning process can be avoided.

Evidence showed that in the implementation of ARMS, neural activity in the primary motor cortex (M1) encodes the movement parameters of direction [85–87], hand position, velocity [88], acceleration [89] and reaching distance [90]. In addition, since proprioceptive feedback is significantly delayed compared to visual feedback, human visual feedback plays an important role in sensory feedback while performing fast ARM [5]. In the proposed model, the regulatory nature of motor planning is based on visual feedback from the hand’s position. In previous researches, movements were planned without considering the environmental effects and sensory feedback, and only by optimizing a cost function [9–15,65,66]. As an (or another) innovation in this study, and in agreement with evidence reported in [5,85–90], regulatory state of ARM is corrected considering the sensory information of the movement.

Recently, it has been shown that the computational mechanism in M1 can be justified due to the optimal feedback control [91,92]. In this study, at the motor command production level, the NPC uses the nature of the feedback control by minimizing output error and MSs’ effort (Eq 10). Thus, the motor command is produced in the motor cortex and applied to the lower levels for implementation. On the other hand, the cerebellum seems to monitor the movement commands and adjusts the motor command to improve the accuracy of the movement [93]. Some researchers have shown that there are some internal models in the cerebellum, which are used in the control of motion [56,94]. Thus, the states of the system can be predicted by considering motor command in a feedforward model and using state variables in the present and the past time. This is completely consistent with the nonlinear predictive nature of the proposed model in this study.

## Conclusion

In this study, we introduced a novel computational model with a hierarchical and modular organization, which is based on recruitment of MSs, for controlling ARM in the frontal planes. To the best of authors’ knowledge this is the first computational model with these features that interacts with the environment, i.e., it describes how the CNS rejects disturbances and controls a combined task based on the control strategy applied for a simple task. The modular organization is used to describe the recruitment of MSs. This organization can explain how the CNS reduces the dimension of the control space in order to simplify motor control procedure. Moreover, inspired by [3,4], we used a hierarchical organization with three levels of motor planning, command production, and motor execution. However, we modified the function of the planning level. In fact, we believe that for the ARM the CNS applies a regulatory and goal-directed approach, and therefore, at the level of motor planning it defines only the desired body or limb states at the starting and final positions while the limb is interacting with the environment (*Fig 1A*). This is a feature that distinguishes this model from other computational models. Therefore, in this model the CNS tracks no desired and predefined trajectory in the joint space. In other words, the joint trajectories during the movement are generated as a result of the natural dynamics of the musculoskeletal system and therefore, the complex trajectory planning procedure is avoided. Since there is no trajectory tracking, it is also possible to execute fast arm reaching motions. On the other hand, for the combined movements, the regulatory input is generated based on the visual and kinematic information in interaction with the environment. At the motor command production level, an NPC determines the RCs of the MSs, while it takes into account the nonlinear nature of the upper limb including the 15 muscles.

At the level of motor execution, a nonlinear model of the upper limb composed of 15 muscles and three DoF in the shoulder joint and one DoF in the elbow joint with moment arms of each muscle is considered in order to represent the performance of the ARMs in the frontal plane, for the first time.

Using this proposed hierarchical and modular computational model, only five RCs of MSs were controlled rather than controlling the 15 muscles individually. This models suggests that possibly the CNS simplifies the control process by reducing the dimension of the control space.

As the next step in our future works, we would like to modify this model and introduce the learning procedure to it. Then, the augmented model will be used to understand the patterns of synergy formation and recruitment during new task learning or re-learning (*e*.*g*., during rehabilitation after stroke).

## Supporting information

S1 Fig The flowchart of force synergy calculation from raw EMG signal. (TIF)

S1 File Appendix A. Data acquisition protocol.
(DOCX)

S2 File Appendix B. Moment arm equations.
(DOCX)

S3 File Muscle Synergies for all 6 subjects. (XLS)

S4 File Subject#1_data. The EMG and position data for different ARMs of Subject #1.
(RAR)

S5 File Subject#2_data. The EMG and position data for different ARMs of Subject #2.
(RAR)

S6 File Subject#3_data. The EMG and position data for different ARMs of Subject #3.
(RAR)

S7 File Subject#4_data. The EMG and position data for different ARMs of Subject #4.
(RAR)

S8 File Subject#5_data. The EMG and position data for different ARMs of Subject #5.
(RAR)

S9 File Subject#6_data. The EMG and position data for different ARMs of Subject #6.
(RAR)
