# Supplementary figures and images for: How does the CNS control arm reaching movements? Introducing a hierarchical nonlinear predictive control organization based on the idea of muscle synergies
Source: PLoS One. 2020 Feb 5;15(2):e0228726. doi: 10.1371/journal.pone.0228726 (PMC7001977; doi:10.1371/journal.pone.0228726)

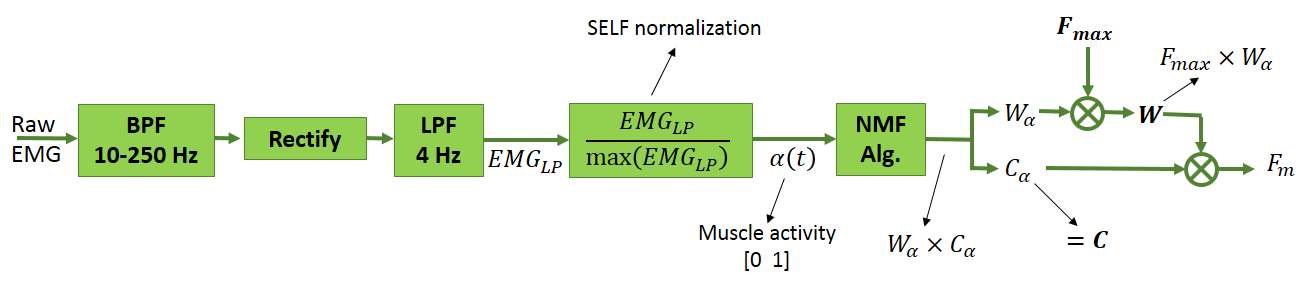

Supplement: S1 Fig — (TIF) [file pone.0228726.s001.tif]
