# Supplementary material for: How does the CNS control arm reaching movements? Introducing a hierarchical nonlinear predictive control organization based on the idea of muscle synergies
Source: PLoS One. 2020 Feb 5;15(2):e0228726. doi: 10.1371/journal.pone.0228726 (PMC7001977; doi:10.1371/journal.pone.0228726)
Supplement: S1 File — Data acquisition protocol. (DOCX) [file pone.0228726.s002.docx]

# Appendix A: Data acquisition protocol

The positions of markers mounted on the shoulder, elbow, and wrist joints were recorded using a 30-Hz Xbox one camera and their three-dimensional coordinates were determined using the code written in visual studio software. The position of the robot handle was recorded with a frequency of 100 Hz and a resolution of 3 mm. The kinematic data were low-pass filtered (FIR filter; 15 Hz cutoff; zero-phase distortion; Matlab fir1 and filtfilt functions) and differentiated to compute tangential velocity. On the other hand, the surface EMG signal of 15 muscles (Biceps, Brachioradialis, Pronator teres, Triceps brachii (lateral and long heads), Deltoid (anterior, middle and posterior), Pectoralis (clavicular and sternal portions), Trapezius (medial and superior), Latissimus dorsi, Teres major, and Infraspinatus) were collected using the myon 320 device at a frequency of 2 kHz. The placement of the electrodes on the skin surface is determined according to the information provided in [1]. To obtain muscle force information, first the EMG signal was band-pass filtered (Butterworth filter, 10-250 Hz cut-off, 2^nd^ order, Matlab fir1, and filtfilt functions). Then, it was digitally full-wave rectified (its absolute value was calculated) and then, integrated over time of 10 milliseconds. Afterward, the envelope of the filtered EMG is calculated by a Butterworth low-pass filtering with a cutoff frequency of 4 Hz and zero-phase distortion. For each movement the onset and offset times were defined respectively as the time where, the tangential velocity profile crossed 5% of its maximum value, and the movement duration (MT) defined as the interval between the onset and offset instants.

The amount of muscle force can be determined using a nonlinear muscle model (for example Hill-type model) as a function of muscle activity. It was shown that in static isometric contractions, there is a linear relationship between muscle forces and smoothed rectified EMG signal [2]. Here, for the sake of simplicity of our numerical calculations (for Force synergies), the muscle force is assumed to be proportional to the muscle activity [2–4]. That is:

$F_{m}=f(\alpha\left( t \right),l_{m},\dot{l_{m}})\cong F_{max}\times\alpha\left( t \right)$ (A1)

Where $\alpha\left( t \right)$ represents muscle activation, $f$ is a nonlinear function, and $F_{max}$ is the maximum muscle force. When all the muscles engaged in the ARM are considered, equation A1 has to be modified to a matrix form, that is, $F_{m}$ will be an $m\times n$ matrix, $F_{max}$ a diagonal $m\times m$ and $\alpha\left( t \right)$ an $m\times n$ matrices, where, $m$ is the number of muscles and $n$ is the number of time samples. ‎S1 Table lists the maximum muscle forces corresponding to the muscles engaged in the ARMs. It should be noticed that $\alpha\left( t \right)$ is normalized and its values belong to [0 1].

1. Maximum force of 15 muscles engaged in the ARMs studies in this research [5,6]

| **No** | **Muscle Name** | **Fmax** | **Based on Reference** |
| --- | --- | --- | --- |
| 1 | biceps brachii | 624.3 | [6] |
| 2 | brachioradialis | 261.3 | [6] |
| 3 | pronator teres | 566.2 | [6] |
| 4 | triceps brachii, lateral head | 624.3 | [6] |
| 5 | triceps brachii, long head | 798.5 | [6] |
| 6 | deltoid, anterior | 1142.6 | [6] |
| 7 | deltoid, middle | 1142.6 | [6] |
| 8 | deltoid, posterior | 259.9 | [6] |
| 9 | pectoralis major, clavicular portion | 364.4 | [6] |
| 10 | pectoralis major, sternal portion | 515.4 | [6] |
| 11 | trapezius medial | 728.4 | [5] |
| 12 | trapezius superior | 728.4 | [5] |
| 13 | latissimus dorsi | 389.1 | [6] |
| 14 | Teres major | 425.4 | [6] |
| 15 | infraspinatus | 1210.8 | [6] |

Since the NMF algorithm calculates a new projection of the data set based on the standard deviations of the variables, therefore a variable with a high standard deviation will have a higher weight in calculation of synergies than a variable with a low standard deviation. By normalizing the data before NMF decomposition, all variables have the same weight in the calculation and thereby, the objective function in NMF algorithm will be optimized more properly.

Here, in extracting the MSs, we used SELF normalization method [7–9] of the data for each muscle (each processed EMG channel) to levelize the standard deviation. Therefore, each channel of the processed EMG data for each subject is normalized to its maximum amplitude across all conditions for that subject. The resulting signal is considered as an approximation for the muscle activity [2,10]. ‎S1 Fig shows the flowchart of the force synergy calculation from the EMG data.

$activation: \alpha\left( t \right)=\frac{{EMG}_{LP\_filtered}}{\max\left( {EMG}_{LP\_filtered} \right)}={EMG}_{SELF-normalized}$ (A2)

1. The flowchart of force synergy calculation from raw EMG signal.

Since the MSs represent the time-invariant and task-independent properties of muscles, in order to avoid outliers, all trials in each experimental condition were aligned based on the movement onset and averaged using the same method reported in the previous studies [11–16]. Therefore, for each task its time duration was normalized to 100% and resampled to 100 samples. Then, the five repetitions of each type of ARMs were averaged over the normalized time into one averaged signal and this signal was used to extract the MSs. Thus, the MSs obtained from NMF algorithm represent the normalized nature of the data.

$\alpha\left( t \right)=W_{\alpha}\times C_{\alpha}$ (A3)

To obtain the numerical values ​​for each muscle force channel, it is necessary to re-adjust MSs’ values ​​according to the maximum values ​​of each muscle force (F_max). Thus, the numerical value of the components of each MS will be obtained with respect to the actual amount of muscle force. Considering Eq. A1, in order to generate actual muscle forces in the proposed model, the extracted MSs from activation data are this time multiplied by the matrix Fmax, as follows:

$F_{m}=W\times C\cong F_{max}\times W_{\alpha}\times C_{\alpha}$ (A4)

$W=F_{max}\times W_{\alpha} , and C\cong C_{\alpha}$ (A5)

Therefore, the MSs, extracted in this way, are groups of muscle forces activated in synchrony with the fixed relative gains as reported in [12,17].

# References

1. Criswell E. Cram’s introduction to surface electromyography. Jones and Bartlett Publishers; 2011.

2. Hof AL. The relationship between electromyogram and muscle force. Sport Sport. 1997;11(3):79–86.

3. Roh J, Rymer WZ, Beer RF. Robustness of muscle synergies underlying three-dimensional force generation at the hand in healthy humans. J Neurophysiol. 2012;107:2123–2142.

4. Holzbaur KRS, Murray WM, Delp SL. A Model of the Upper Extremity for Simulating Musculoskeletal Surgery and Analyzing Neuromuscular Control. Ann Biomed Eng. 2005;3:829–840.

5. Wochatz M, Rabe S, Wolter M, Engel T, Mueller S, Mayer F. Muscle activity of upper and lower trapezius and serratus anterior during unloaded and maximal loaded shoulder flexion and extension. Int Biomech. 2017;4(2):68–76.

6. Steele KM, Tresch MC, Perreault EJ. The number and choice of muscles impact the results ofmuscle synergy analyses. FrontComputNeurosci. 2013;7(105):1–9.

7. Cheung VCK, D’Avella A, Bizzi E. Adjustments of Motor Pattern for Load Compensation Via Modulated Activations of Muscle Synergies During Natural Behaviors. J Neurophysiol. 2009;101:1235–57.

8. Cheung VCK, Piron L, Agostini M, Silvoni S, Turolla A, Bizzi E. Stability of muscle synergies for voluntary actions after cortical stroke in humans. Proc Natl Acad Sci U S A. 2009;106(46):19563–19568.

9. Cheung VCK, Turolla A, Agostini M, Silvoni S, Bennis C, Kasi P. Muscle synergy patterns as physiological markers of motor cortical damage. Proc Natl Acad Sci U S A. 2012;109(36):14652–6.

10. Eskandari AH, Sedaghat-Nejad E, Rashedi E, Sedighi A, Arjmand N, Parnianpour M. The effect of parameters of equilibrium-based 3-D biomechanical models on extracted muscle synergies during isometric lumbar exertion. J Biomech. 2016;49(August):967–73.

11. Ting LH, Macpherson JM. A Limited Set of Muscle Synergies for Force Control During a Postural Task. J Neurophysiol. 2005;93:609–13.

12. Torres-oviedo G, Macpherson JM, Ting LH, Macpherson JM, Ting LH. Muscle Synergy Organization Is Robust Across a Variety of Postural Perturbations. J Neurophysiol. 2006;96:1530–46.

13. D’Avella A, Portone A, Fernandez L, Lacquaniti F. Control of Fast-Reaching Movements by Muscle Synergy Combinations. J Neurosci [Internet]. 2006;26(30):7791–810. Available from: http://www.jneurosci.org/cgi/doi/10.1523/JNEUROSCI.0830-06.2006

14. Russo M, Andola MD, Portone A, Lacquaniti F, D’Avella A. Dimensionality of joint torques and muscle patterns for reaching. Front Comput Neurosci. 2014;8(24):1–21.

15. Delis I, Panzeri S, Pozzo T, Berret B. A unifying model of concurrent spatial and temporal modularity in muscle activity. J Neurophysiol [Internet]. 2014;111(3):675–93. Available from: http://www.ncbi.nlm.nih.gov/pubmed/24089400

16. Hilt PM, Delis I, Pozzo T, Berret B. Space-by-Time Modular Decomposition Effectively Describes Whole-Body Muscle Activity During Upright Reaching in Various Directions. Front Comput Neurosci. 2018;12(April):1–19.

17. Ting LH. Dimensional reduction in sensorimotor systems: A framework for understanding muscle coordination of posture. Prog Brain Res. 2007;165:299–321.
