# Supplementary material for: How does the CNS control arm reaching movements? Introducing a hierarchical nonlinear predictive control organization based on the idea of muscle synergies
Source: PLoS One. 2020 Feb 5;15(2):e0228726. doi: 10.1371/journal.pone.0228726 (PMC7001977; doi:10.1371/journal.pone.0228726)
Supplement: S2 File — Moment arm equations. (DOCX) [file pone.0228726.s003.docx]

# Appendix B: Moment arm equations

To the author’s best of knowledge, there has been no integrated model for calculating the moment arm of 15 muscles involved in ARM with four DoF in the frontal plane. Therefore, as another innovation in this study, according to the previous studies and measurements, the values of the moment arm for each muscle is defined as a polynomial function of joint angles leading to its movement. Non-zero elements of the moment arm matrix in cm is defined as the following relationships with respect to joint angles:

S2 Table. The non-zero elements of the moment arm matrix in cm

|  | According to reference | Moment arm equation  $\theta,\eta,\zeta,\phi$ |
| --- | --- | --- |
| (B1) | [1] | $M_{arm}\left( 1,4 \right)=M_{arm}\left( 1,5 \right)=0.0057 \theta^{2}-0.0313 \theta+0.0451$ |
| (B2) | [1] | $M_{arm}\left( 1,6 \right)=M_{arm}\left( 1,7 \right)=M_{arm}\left( 1,8 \right)= 0.0004 \theta^{2}-0.0103 \theta+0.0362$ |
| (B3) | [2] | $M_{arm}(1,11)=1.8$ |
| (B4) | [2] | $M_{arm}(1,12)=2.7$ |
| (B5) | [1] | $M_{arm}\left( 1,13 \right)=-0.0026 \theta^{2}+0.0058 \theta+0.0538$ |
| (B6) | [1] | $M_{arm}\left( 1,14 \right)=-0.0081 \theta^{2}+0.0509 \theta+0.0722$ |
| (B7) | [1] | $M_{arm}\left( 2,9 \right)=M_{arm}\left( 2,10 \right)=0.0041 \eta^{2}-0.0382 \eta+0.0924$ |
| (B8) | [1] | $M_{arm}\left( 2,13 \right)=0.0158 \eta^{2}-0.1142 \eta+0.2335$ |
| (B9) | [1] | $M_{arm}\left( 2,14 \right)=0.0025 \eta^{2}-0.0151 \eta+0.0284$ |
| (B10) | [3] | $M_{arm}\left( 3,6 \right)=M_{arm}\left( 3,13 \right)=M_{arm}\left( 3,14 \right)=-0.3839 \zeta+0.7$ |
| (B11) | [3] | $M_{arm}\left( 3,15 \right)=0.3839 \zeta+0.7$ |
| (B12) | [1] | $M_{arm}\left( 4,1 \right)=0.0065 \phi^{2}-0.0362 \phi+0.0346$ |
| (B13) | [1] | $M_{arm}\left( 4,2 \right)=0.0108 \phi^{2}-0.0573 \phi+0.0683$ |
| (B14) | [4] | $M_{arm}\left( 4,3 \right)=1.7$ |
| (B15) | [1] | $M_{arm}\left( 4,4 \right)=M_{arm}\left( 4,5 \right)=-0.001 \phi^{2}+0.0011 \phi-0.0068$ |

# References

1. Michilsens F, Vereecke EE, Aou KD, Aerts P. Muscle moment arms and function of the siamang forelimb during brachiation. J Anat. 2010;217:521–35.

2. Johnson GR, Pandyan AD. The activity in the three regions of the trapezius under controlled loading conditions - An experimental and modelling study. Clin Biomech. 2005;20(2):155–61.

3. Toledo JM, Ribeiro DC, Loss JF. Mechanical criteria for progression in internal and external rotation exercises of the shoulder in the sagittal plane. Rev Bras Fisioter [Internet]. 2007;11(1):49–56. Available from: http://www.scielo.br/scielo.php?script=sci_arttext&pid=S1413-35552007000100009%5Cnhttp://www.scielo.br/scielo.php?script=sci_pdf&pid=S1413-35552007000100009&lng=en&nrm=iso&tlng=pt

4. Ramsay JW, Hunter B V, Gonzalez R V. Muscle moment arm and normalized moment contributions as reference data for musculoskeletal elbow and wrist joint models. J Biomech. 2009;42:463–73.
